# Can Machine Learning Provide Understanding? How Cosmologists Use Machine Learning to Understand Observations of the Universe

**DOI:** 10.1007/s10670-021-00434-5

**Published:** 2021-07-21

**Authors:** Helen Meskhidze

**Affiliations:** Department of Logic and Philosophy of Science, University of California, Irvine, Irvine, USA

## Abstract

The increasing precision of observations of the large-scale structure of the universe has created a problem for simulators: running the simulations necessary to interpret these observations has become impractical. Simulators have thus turned to machine learning (ML) algorithms instead. Though ML decreases computational expense, one might be worried about the use of ML for scientific investigations: How can algorithms that have repeatedly been described as black-boxes deliver scientific understanding? In this paper, I investigate how cosmologists employ ML, arguing that in this context, ML algorithms should not be considered black-boxes and can deliver genuine scientific understanding. Accordingly, understanding the methodological role of ML algorithms is crucial to understanding the types of questions they are capable of, and ought to be responsible for, answering.

## Introduction

The scientific value of cosmological observations, observations of the large-scale structure of the universe that are used to infer various cosmological parameters, hinges critically on the simulations used to interpret them.^1^ The increasing precision of the observations, however, has created a problem for simulators: the simulations necessary to interpret the observations have become too large and too complex for meaningful analyses to be performed on or with them. Specifically, the parameter spaces the simulations investigate are enormous and the simulations themselves exhibit non-linear behavior. Running these sorts of simulations has become impractical because of the associated computational expense. To address this difficulty, simulators have turned to machine learning (ML) algorithms. ML algorithms offer substantial reductions in computational costs.

Though they reduce computational costs, one might be worried about the use of machine learning for scientific investigations: How can algorithms that have repeatedly been described, sometimes by their own developers, as black-boxes deliver scientific understanding?^2^ One group of cosmologists that uses Gaussian processes, for example, describes their machine learning algorithm as an “infinitely fast black-box oracle” (Lawrence et al. 2010, 50). Another group that uses artificial neural networks writes, “One might argue that a machine-learning approach to determine the non-linear response from varying parameter settings is a rather black-box approach that goes against the traditional approach to spectra: based on scientific understanding and physics” (Agarwal et al. 2012, 1410). However, they justify this new approach pragmatically, citing the “impending flood of new data from upcoming surveys” (2012, 1410). While the pragmatic benefits of using ML are clear, the worry remains that in employing ML, investigators are sacrificing scientific understanding for the sake of reductions in computational expense. This worry is shared by many who use ML methods and warrants serious consideration.

In this paper, I investigate the use of two machine learning algorithms in cosmology: the use of Gaussian processes by Heitmann et al. (2010) and the use of artificial neural networks by Agarwal et al. (2012).^3^ I begin by first drawing a distinction between black-boxes themselves and black-boxing as a methodology, or “the method of ignoration” as I will call it. I then claim that in the context of this case study, ML algorithms ought not be considered black-boxes but as part of a larger methodology that uses the method of ignoration. More specifically, I argue that machine learning algorithms allow cosmologists to use the method of ignoration on their underlying simulations in order to explore statistical relationships between the responses of the simulations to various inputs. I take my argument to establish that whatever understanding is gained by N-body simulations, the same can be gained by these ML algorithms. This case study serves as a proof of concept—that ML can deliver genuine scientific understanding and so the use of ML is not in principle incompatible with scientific understanding. It also highlights a larger lesson: the context in which ML algorithms are employed is crucial to understanding the types of questions they are capable of, and ought to be responsible for, answering.

I begin by distinguishing two often conflated senses of the term “black-box,” both of which will be informative in analyzing contemporary machine learning algorithms. The remainder of the paper is devoted to exploring cosmological applications. With this in mind, I begin Sect. 3 by presenting some of the physics behind investigations of the large-scale structure of the universe. I then describe the simulations cosmologists use as well as two machine learning methods. In Sect. 4, I discuss whether such methods ought to be understood as black-boxes and investigate how they provide cosmologists understanding. Section 5 offers a brief conclusion.

## Black-Boxes and the Method of Ignoration

### Black-Boxes

In Vitold Belevitch’s summary of the history of circuit theory, he claims that the term black-box originated in the context of electrical engineering. Specifically, Belevitch writes that the term was first used in 1921 in reference to two-port networks (circuits with two pairs of external terminals; (Belevitch 1962, 848–849)). Such circuits can be completely characterized with four numbers: the voltage and current across the two ports.

The term “black-box” then crossed disciplines from electrical engineering into cybernetics, or the science of communications and control of life and machines. W. Ross Ashby’s 1956 *Introduction to Cybernetics* includes an entire section titled “The Black Box” in which Ashby writes, “In our daily lives we are confronted at every turn with systems whose internal mechanisms are not fully open to inspection, and which must be treated by the methods appropriate to the Black Box” (Ashby 1956, 86). In brief, this early literature uses the term “black-box” to designate an object whose internal mechanisms cannot be seen, but that one learns to control by studying its input/output relations.

### The Method of Ignoration

A second strand emerged in the literature on black-boxes which referred not to black-boxes as objects, but to black-boxing as a methodology—or, treating something as a black-box. This concept has its roots in a much older text: William Thomson (later, Lord Kelvin) and Peter Tait’s *Treatise on Natural Philosophy*. They mention the method only briefly, calling it the method of ignoration, but James Clerk Maxwell elaborates on the idea in his review of their treatise.^4^ He writes:

In the cases to which the method of ignoration is applied there are certain variables of the system such that neither the kinetic nor the potential energy of the system depends on the values of these variables, though of course the kinetic energy depends on their momenta and velocities. The motion of the rest of the system cannot in any way depend on the particular values of these variables, and therefore the particular values of these variables cannot be ascertained by means of any observation of the motion of the rest of the system. *We have therefore no right, from such observations, to assign to them any particular values, and the only scientific way of dealing with them is to ignore them.* (1879, 215; emphasis my own).

I take Maxwell to be saying that if one believes the object of investigation is insensitive to the values of certain variables, then one cannot specify any such values. Thus, the method of ignoration suggests that one ought to ignore these variables.

Though Maxwell’s terminology did not endure, the idea behind the method of ignoration did. Consider, for example, the following passage from John Blatt and Victor Weisskopf’s book *Theoretical Nuclear Physics*:

In the language of electrical engineering, we treat the compound nucleus as a “black-box” with *N* terminals, one for each channel. The voltage and current at each terminal are the only quantities of interest for the behavior of the “black-box” toward the outside. In particular, two different black boxes which give rise to the same currents and voltages at the terminals are equivalent for our purposes. (1952, 543–544)

Though like Ashby, Blatt and Weisskopf draw upon the electrical analogy, their use of the term black-box matches what Thomson and Tait meant by the method of ignoration: it involves deliberately ignoring mechanisms deemed to be insignificant to the object of investigation. Though subsequent to Maxwell’s writings, this methodology has gone under the name black-boxing, I will continue to refer to it as the method of ignoration to avoid confusion with black-boxes themselves.

To better understand this distinction between black-boxes and the method of ignoration, it will be informative to connect the above historical discussion to a contemporary account. In Michael Strevens’s paper “Special-Science Autonomy and the Division of Labor” (2016), he discusses how an investigator might choose to ignore processes that are at the same level of detail or at lower levels of detail as the phenomenon of interest. I understand him to be describing what I have called the method of ignoration. As Strevens explains, there are two reasons an investigator might be justified in employing the method of ignoration: first, if the details of the lower-level do not matter to the phenomenon they are investigating. This could be because the object of investigation is emergent from, and thus insensitive to, these details or because the problem is sufficiently modular. Second, if the scientific division of labor excuses the investigator from supplying an understanding of the ignored processes. This might be the case if it is the job of another discipline to supply the details of these processes (2016, 166–172). Overall, the knowledge that the scientist already has in such a case is different from the case where she confronts a black-box. In Strevens’s case, the scientist already has theoretical knowledge and knows what variables matter to the investigation. When confronting a black-box, the scientist is trying to develop this theoretical knowledge.

To summarize, I have pulled apart two ideas in the above discussion: black-boxes, understood as objects, and the method of ignoration, understood as a methodology. This distinction will be important as we consider whether machine learning algorithms are themselves black-boxes, if they use the method of ignoration, or neither. Before addressing this question, I first turn to presenting the theoretical background behind cosmological simulations.

## Inferring Cosmological Parameters from the Large-Scale Structure of the Universe

### The Theoretical Background

Cosmologists have been interested in the large-scale distribution of matter in the universe since the early twentieth century.^5^ Hubble’s observations in the 1930s indicated that, at sufficiently large scales, galaxies are distributed homogeneously in the universe. On smaller scales, however, Hubble showed that planets, stars, galaxies, and even groups of galaxies exhibit clustering, forming so-called “structures.” Attention was next turned to understanding Hubble’s observations: Could such observations be predicted directly from (gravitational) theory or could they guide future theoretical research?

Given the enormous length and time scales involved, the basic theory behind large-scale structure formation is remarkably simple.^6^ One models the (dark) matter in the universe as a perfect and homogeneous fluid and gravity with a Newtonian gravitational potential. The creation of structure requires deviations from homogeneity, so one introduces small density perturbations and studies their evolution. The method outlined so far is applicable as long as the density fluctuations remain small. As the system is evolved forward and the perturbations grow to be larger (on the order of the background fluid density or greater), however, this simple linear theory is no longer quantitatively useful. To be able to compare theoretical predictions with observations, cosmologists need to study these larger perturbations and the structures that they seed. Thus, they turn to another method: simulations.

### Investigating the Large-Scale Structure of the Universe with Simulations

The simulations used to investigate the large-scale structure of the universe begin much like the theory described above. One first assumes that the (dark) matter in the universe can be modeled as a perfect fluid. Because these are computer simulations, they represent this perfect fluid as a discrete set of particles (N-bodies) interacting via Newtonian gravitational forces. For this reason, such simulations are called “N-body simulations.” Importantly, as Coles and Lucchin note, though they employ discrete particles, “these techniques are not intended to represent the motion of a discrete set of particles. The particle configuration is itself an approximation to a fluid” (2002, 305). Indeed, the particles themselves are not meant to be representations of real, physical particles but rather each particle “represent[s] a phase space patch covering a vast number of individual [dark matter] particles” (Tulin and Yu 2018, 26).

In this shift to simulations, cosmologists are no longer asking *if* gravitational theory can predict structure formation—this question has already been answered affirmatively. Instead, they are using simulations to investigate the statistical distributions of matter that different combinations of cosmological parameters give rise to.^7^ The values of these cosmological parameters are not typically constrained directly by observations. Instead, observations of the statistical distributions of matter (i.e., the matter power spectrum) provide constraints to many of these parameters at once. Thus, cosmologists require large data sets from simulations that vary the parameters of interest to compare to observations and infer the values of the cosmological parameters instantiated in the universe.

The challenge of N-body simulations comes not from the theory underlying the simulations, but from the execution of the simulations. The first difficulty is that each simulation employs millions of particles (e.g., Heitmann et al. 2010 employ over 16 million particles), so calculating the pairwise gravitational forces between particles, summing the total forces, and evolving the entire system forward is computationally expensive. The second difficulty associated with N-body simulations is that using these simulations to infer cosmological parameters requires having comprehensive coverage of the parameter space of cosmological parameters. This is because the standard method for inferring cosmological parameters from such simulations is to use Markov Chain Monte Carlo (MCMC) analysis. MCMC analysis is a method of Bayesian inference that allows researchers to sample from a probability distribution. In this case, MCMC analysis is used to sample from a probability distribution over cosmological parameters and determine what parameter values are instantiated in observations. When this requirement for comprehensive coverage of the parameter space is coupled with the expense of running each simulation, the impracticality of the task becomes obvious.

### What do Cosmologists Learn from N-Body Simulations?

Researchers conducting N-body simulations acknowledge the limits of their gravity-only simulations. Heitmann and her collaborators note, for example, that at sufficiently small length scales, additional physics beyond mere gravitational interactions will be needed for accurate calculations (e.g. gas dynamics and feedback effects; 2010, 105–107). Nonetheless, N-body simulations are a good approximation and useful for studying the effects of changing the cosmological parameters on large-scale structure formation.

Given that N-body simulations leave out what is known to be relevant physics on small scales, one may wonder what cosmologists are trying to learn with such simulations. In this context, cosmologists are clearly not asking the question “When all our best models of the relevant physics have been included, do we get a universe like ours?” Their investigations cannot be aimed at this question as their simulations leave out large domains of relevant physics. The framework of minimalist idealization (or minimal conditions modelling) can help clarify the situation. Weisberg describes minimalist idealization as “the practice of constructing and studying theoretical models that include only the core causal factors which give rise to a phenomenon” (Weisberg 2007, 642). O’Connor writes similarly that minimal conditions modeling identifies reasonable, minimal conditions for a phenomenon to arise (2017, 7). Perhaps, then, N-body simulations should be understood as a minimalist idealization—as modeling the minimal conditions for large-scale structure formation. N-body simulations *do* show that gravitational force is a minimal causal variable in producing the large-scale distribution of matter, but it also seems clear that this is not all cosmologists are learning from such simulations.

I suggested above that such simulations are designed to answer questions such as: “What would the statistical distribution of matter in the universe be if these were the true values of the cosmological parameters, assuming some particular cosmological model?” To appreciate the importance of this question, consider the role of such simulations in cosmologists’ larger research programs. The results of N-body simulations are often compared to cosmologists’ ever-improving observations of the statistical distribution of matter in the universe.The interplay between observations and theory/simulations points us towards the role of such simulations: considered together, observations and simulations serve as tests of different instantiations of cosmological parameters.

We can also ask whether and how such simulations are explanatory. To address this question, consider a distinction Batterman draws between what he calls type (i) and type (ii) why questions. Type (i) why questions ask why a phenomenon occurred in some particular circumstance while type (ii) why questions ask why phenomena of this general type occur across a variety of circumstances (1992, 332). In a later paper, Batterman and Rice (2014) argue that the explanations provided by minimal models fit within this second why question and are distinct from various others kinds of explanations (e.g., causal, mechanical, etc.) discussed in the philosophy of science literature. They claim that minimal models are explanatory insofar as they provide a story about why a class of systems all exhibit some large-scale behavior.

I argue that the simulations discussed above are actually answering both types of questions. The type (i) why question is “Why does our universe have the particular statistical distribution of matter that it does?” The answer these N-body simulations give would include the values of the cosmological parameters in the cosmological model being tested. The type (ii) why question is “Why does the universe exhibit structures across a variety of cosmological parameters?” This question (which is closer to one minimal conditions modeling is meant to answer) could then be answered by both linear theory and N-body simulations. They would both point to gravitational forces acting on small perturbations to bring about clustering behavior.

Ultimately, these N-body simulations do address the minimal conditions needed for structure formation. More importantly, however, coupled with observations, they serve as tests of instantiations of various cosmological parameters. For such simulations to fulfill this role requires that they be at least as precise as the observations they are being compared to. Considering the huge computational expense involved in running these simulations, it is unsurprising that cosmologists are looking for new methods to employ in these contexts.

### Investigating the Large-Scale Structure of the Universe with Machine Learning

ML has a long history of use in astronomy and cosmology. Some of the first uses included scheduling observation time and performing adaptive optics for array telescopes (see Serra-Ricart et al. 1994, for a review of uses in the early 1990s). Contemporary uses of ML range from identifying structure in simulations to interpreting observations of the cosmic microwave background.^8^ The role of ML in the next decade of cosmology was the topic of a recent white paper submitted as part of the Astro2020 Decadel Survey on Astronomy and Astrophysics organized by the National Academy of Sciences. There, Ntampaka and collaborators argued that the upcoming “era of unprecedented data volumes” (2019, 3) in cosmology provides rich opportunities to employ ML techniques. They further argue that cosmology is uniquely positioned not only to benefit from advances in ML, but to itself provide “opportunities for breakthroughs in the fundamental understanding of ML” (Ntampaka et al. 2019, 5). They consider the corresponding “temptation to choose expediency over understanding” (2019, 3) but outline some methods for improving the interpretability of ML. It is with these same worries and goals that I have chosen to focus on a cosmological case study in this paper.

The case study presented here uses ML to address the second of the two sources of computational expense in the context of N-body simulations. Recall, from Sect. 3.2, that the first source is the number of particles needed for any individual N-body simulation while the second is the number of simulations needed for MCMC analysis. Cosmologists have begun using ML methods like Gaussian processes and artificial neural networks to quickly fill in the relevant parameter space using a limited number of simulations, thus addressing the second source of computational expense. One of the first groups to employ ML to study large-scale structure formation was Katrin Heitmann’s research team. They call their methodology “emulation,” describing it as a “generalized interpolation method capable of yielding very accurate predictions” (Heitmann et al. 2009, 2). But what is an emulator, how is it different from a simulation, and how does it use machine learning to reduce computational expense?

Developing an emulator requires: (i) building a training set (often just a collection of simulation results), (ii) regressing for analytic functions that mimic the training set data, and (iii) evaluating those functions at the desired interpolation points while accounting for interpolation error Schneider et al. (2011); Kern et al. (2017).^9^ As Kern et al. note, “The emulator itself is then just the collection of these functions, which describe the overall behavior of our simulation” (2017, 2–3). Emulators do not include physical laws or principles. Rather, they statistically characterize the space of simulation results and allow for sophisticated interpolation.

Below, I present the methodology used to construct two emulators, the Cosmic Emulator and Pkann. I have chosen these two emulators for a variety of reasons. First, because of the relative simplicity of the goal of the two emulators—to fill in the parameter space needed for MCMC analysis. This simple goal makes them a valuable case study to investigate the role of emulators in broader research contexts and to provide a proof of concept that ML can deliver scientific understanding. Second, because both research groups express skepticism about the ability of their emulators to deliver scientific understanding (as discussed in Sect. 1). I will argue that when understood in the larger research context, these emulators can overcome the worries expressed by their developers and provide explanations.^10^

#### The Cosmic Emulator

The construction of Heitmann’s emulator, the so-called Cosmic Emulator, proceeds according to the three steps outlined above. Heitmann et al. begin with a five-dimensional parameter space, with each dimension corresponding to each of the five cosmological parameters they are investigating. They then decide on a methodology to sample the parameter space and run the appropriate simulations. They employ Symmetric optimal Latin Hypercube (SLH) sampling, a sampling method that imposes good filling and sampling of the parameter space and is thought to be most appropriate when one is ignorant of functional variation across the parameter space (Habib et al. 2007, 5). Using SLH sampling, Heitmann et al. find that only 37 cosmological simulations are necessary to train their emulator. In other words, they only need 37 points in the five-dimensional parameter space (Heitmann et al. 2009, 163).

Having built their training set, Heitmann and her collaborators decide to use Gaussian Process (GP) modeling to interpolate amongst the simulations runs. GP modeling works by finding the function that best characterizes the data through Bayesian inference. As noted by Mohammadi et al., GPs have several advantages. They can be used to fit any smooth, continuous function and they are considered “non-parametric,” meaning “no strong assumptions about the form of the underlying function are required” (Mohammadi et al. 2018, 2). This makes them especially compatible with the sampling methodology employed for the Cosmic Emulator.

Once the emulator is trained, the final step in the process is to test the emulator. Heitmann et al. consider a mock data set of 10 test cosmological models and find that emulation reproduces the nonlinear matter power spectrum to within 1% accuracy (2009, 167). Ultimately, the fully trained emulator allows an investigator to use MCMC analysis to infer the values for various cosmological parameters that would have given rise to a particular observation.

#### PkANN

Artificial neural networks (ANNs) are another method of machine learning that has been employed by cosmologists. In a series of two papers, Agarwal et al. (2012, 2014) present PkANN, an ANN-based emulator.^11^ The main advantage ANNs have over GPs in this context is their ability to cover a broader parameter space, but the drawback is that ANNs require a much larger training set of simulations.

Agarwal’s methodology, like Heitmann’s, begins with LH sampling. Then, instead of a GP, they train an ANN on this simulation set and evaluate the ANN’s accuracy. Fundamentally, an ANN consists of interconnected nodes which can be thought of as artificial neurons with activation functions. These activation functions map the input the node receives to its output. These nodes are then arranged in “layers” and allowed to communicate, to transport their output, to nodes in the next layer. The connections linking nodes do not merely transmit the information; they also multiply the output of the previous node by a “weight” as it travels along the connection to the next node. Adjusting these weights to get better results constitutes the “training” of an ANN. Though one can in principle perform this training by hand (adjusting the weights to match the network’s output to the desired output), the sheer number of weights in an ANN often prevents one from being able to do so meaningfully. Instead, the ANN typically adjusts the weights itself, a process referred to as “learning.” Training an ANN in this way requires having a labeled training set. The ANN then compares its own output to a known answer from the training set and quantifies the difference with a “cost function.” The ANN then shifts the weights in whatever direction is required to get a better evaluation from the cost function. In sum, whereas Gaussian processes use Bayesian inference to find the function that best characterizes the data, ANNs are essentially trained to solve a calculus problem: to minimize their cost function by determining the necessary weight parameters for their model.^12^

Once they have a trained ANN, Agarwal et al. use it to fill in the required parameter space. They then use MCMC analysis to infer the values of various cosmological parameters. Their fully trained emulator outperforms the Cosmic Emulator but requires an order of magnitude more simulation runs for its training set.

## What do Cosmologists Learn Using Machine Learning?

I claimed above that when N-body simulations are combined with observations, they provide understanding insofar as they serve as minimal models for structure formation and as tests of various cosmological models. From the above description of the Cosmic Emulator, it is obvious that emulators do not themselves model the relevant physics. We are thus faced again with the question posed at the outset: How can algorithms that do not model physical principles and that have repeatedly been described as black-boxes deliver scientific understanding? In my answer to this question, I will draw on the distinction detailed in Sect. 2 between black-boxes and the method of ignoration, and claim that machine learning algorithms actually allow the investigator to employ the method of ignoration on the underlying simulations. Their scientific value, I will argue, hinges on this distinction.

In Sect. 2, I described a black-box as something whose inner mechanisms are unknown to the investigator. On this description, one might think that all computer algorithms and simulations, including machine learning algorithms, are black-boxes. After all, one might claim, who really knows what goes on inside a computer? The argument that all computer algorithms are black-boxes was addressed and rejected in early discussions of the role of computers in scientific investigations. In an article written for* Scientific American *in 1966, computer scientist Anthony Oettinger dismisses the idea that an investigator must know all the inner workings of a computer to understand an algorithm. Instead, he discusses how the role that a computer algorithm plays in a particular investigation informs its (potential) status as a black-box. He distinguishes between computer algorithms that are functionally representative and those that are structurally representative, claiming that only functionally representative algorithms are black-boxes. Functionally representative algorithms mimic the desired input-output relation without including the physical principles behind these relations, whereas structural representations do rely on physical principles and, consequently, deliver scientific understanding. Though I think this account can be developed even further (and do so below), I agree with Oettinger’s claim that not all algorithms are black-boxes and, more importantly, that the role an algorithm plays in an investigation critically informs whether it should be understood as a black-box.

To develop Oettinger’s account further, recall Strevens’s discussion of what I have termed the method of ignoration. He claimed that an investigator might be justified in ignoring certain processes if the problem they are investigating is sufficiently modular or if the phenomenon emerges from lower-level processes (2016, 166–172). Thus, what Oettinger has called a functional representation is not necessarily a black-box; sometimes, I claim, it is actually an employment of the method of ignoration. In other words, it might be the case that scientists are functionally modeling a system because they do not know what processes are important. In this case, the scientists are using both a functional model and a black-box. Importantly, however, they might know what processes are important but still model the system functionally (for, e.g., convenience or simplicity). In such a case, they would be employing the method of ignoration. Both cases are examples of functional modeling but the scientists’ epistemic positions with respect to the system being investigated are radically different.

That functional representations can be either black-boxes or uses of the method of ignoration is what has led to misunderstanding. The machine learning algorithms described in this paper are indeed functional representations, but they are not black-boxes; they are uses of the method of ignoration. Cosmologists have modeled the underlying relations structurally (i.e., physically) with cosmological N-body simulations. They have then trained machine learning algorithms (GPs and ANNs) on these simulation results to explore, and perhaps exploit, emergent statistical relationships without having to continue running computationally expensive N-body simulations. Thus, emulators do ignore the underlying physical mechanisms and employ the method of ignoration, but they are justified in doing so because they are exploring emergent statistical relations.

Naturally, using machine learning algorithms that do not model physical laws has limitations. As discussed in Sect. 3.3, cosmological N-body simulations can answer Batterman’s type (ii) why questions: why phenomena of this general type occur across a variety of circumstances. As minimal models of structure formation, they allow one to abstract away from any details of particular cosmological models, and, in doing so, reveal patterns evident across various instantiations of cosmological parameters. Machine learning algorithms exploit these patterns. This means, however, that machine learning algorithms cannot answer Batterman’s type (ii) why questions. If one is interested in these questions, one ought to consult the underlying N-body simulations. Though they cannot answer type (ii) why questions, machine learning algorithms can answer type (i) why questions: why, for example, our universe has the particular distribution of matter it does. By filling out the parameter space of interest, such methods can point cosmologists to the relevant values of the cosmological parameters that led to a particular distribution of matter. Put differently, these ML algorithms are just as explanatory as their underlying N-body simulations with respect to these type (i) why questions.

Discussion of the explanatory goals of ML can also be cast using Kathleen Creel’s tripartite distinction between functional, structural, and run transparency (2020).^13^ Even if a code exhibits opacity of one of these three types, Creel argues that the code may nonetheless be partially transparent. This gradation of transparency allows her to make sense of recent efforts by computer scientists to increase the transparency of machine learning algorithms.

On Creel’s account, functional transparency is “knowledge of the algorithmic functioning of the whole” (2020, 573), while structural transparency requires that one know *how* a particular algorithm is instantiated in the code. Creel highlights that structural transparency does not necessarily require a step-wise analysis of the code, but rather an understanding of the relations amongst the subcomponents (2020, 578). Run transparency requires knowledge of how the program was run in a particular instance—e.g., the hardware it was run on or (if applicable) the training data used (2020, 580). Importantly, Creel argues that the three types of transparency “are dissociable: each can be exhibited without requiring any of the others” (2020, 581).

In the case of the Cosmic Emulator and PkANN, functional transparency only requires that we know the algorithm instantiated: Gaussian process modeling and artificial neural networks respectively. We presume that the developers of these algorithms understand *how* the code instantiates the algorithms, thus establishing their structural transparency. The most contentious type of transparency in this case is thus run transparency—especially with respect to the training data required. For instance, one might question whether 37 cosmological simulations are sufficient to reliably train the Cosmic Emulator or whether the SLH sampling method is appropriate. However, any such critiques would require further evidence, a demonstration of where the emulator fails, or an argument as to why some alternative sampling technique is superior. Until such evidence is provided, I argue that the emulators exhibit all of the types of transparency outlined by Creel.

To be clear, I am not arguing that machine learning algorithms can provide understanding in all contexts nor do I take myself to have outlined sufficient conditions for ML algorithms to be explanatory. Rather, I hope to have provided an example demonstrating that ML is not in principle incapable of providing scientific understanding as well as to have shown the importance of asking such questions while also considering the broader methodology being employed. In other contexts, contexts in which they are used to explore functional relations and their use goes beyond exploring the statistical relationships of a structural model, such methods might rightly be described as black-boxes and one might rightly be worried about their ability to develop understanding. In particular, in contexts where a ML algorithm or an ANN is answering causal questions and there are no underlying structural simulations to appeal to, one might be rightly worried about them being black-boxes.^14^

## Conclusion

In this paper, I hope to have addressed the worry about the ability for what seem like black-boxes to increase scientific understanding. Some cosmologists have expressed this worry in employing methods like machine learning. In describing the goals of machine learning algorithms in cosmology, I have argued that it is a mistake, at least in this context, to understand these methods as black-boxes. Rather, these methods develop scientific understanding by enabling an investigator to employ the method of ignoration with respect to their underlying simulations. Naturally, there are other contexts in which machine learning algorithms *are* black-boxes themselves, and their use in such contexts deserves careful treatment. Understanding the context ML algorithms like Gaussian processes and ANNs are employed in is crucial to understanding the role they play in the investigation and the types of questions they are capable of, and ought to be responsible for, answering.

## Footnotes

1 Throughout this paper, when I refer to cosmological simulations, I mean only N-body simulations that describe the large-scale structure of the universe and are used to infer cosmological parameters (for more about such simulations, see Sects. 3.1–3.2). Of course, there are many other types of cosmological simulations for various purposes, but I do not consider those here.

2 I use the phrase “scientific understanding” here to capture the general idea of a scientist knowing why something, or a class of things, is the case. Sects. 3.3 and 4 detail the why questions being asked (and answered) more precisely.

3 I have chosen cosmology for this case study as it provides a context in which ML is being used but contextual values (moral, political, personal, social, etc.) are unlikely to be relevant and the explanations desired are solely for scientific purposes.

4 I have David Gooding’s “Philosophy and Science: The Black Box Again” (1974) to thank for the connection between the method of ignoration and black-boxing as a methodology.

5 Much of this exposition follows Peebles’ introduction (1980, 3–36).

6 For a detailed description of the methodology, see Coles and Lucchin (2002, 205–262), Peter and Uzan (2013, 238–246), and Brandenberger (2004).

7 In Heitmann et al. (2009, 2010), the cosmological parameters investigated are: (1) The density of baryonic matter in the universe, (2) the total matter density in the universe, (3) the equation of state for dark energy, (4) the scalar spectral index which describes the scale-dependence of the initial fluctuations, and (5) the amplitude of the fluctuations. Agarwal et al. (2012, 2014) add (6) the sum of the three neutrino masses and (7) the desired redshift.

8 See He et al. (2019) for discussion of the various uses of ML and https://github.com/georgestein/ml-in-cosmology for a comprehensive and up-to-date list of references.

9 There have been a number of projects employing emulators in cosmology/astrophysics (e.g., PICO, cosmopp, CFHTemu1, Aemulus) as well as outside astrophysical contexts (see Kennedy et al. 2006). Despite the diversity of the problems they are tackling, the emulators used by these groups all share the three-step methodology outlined here.

10 I would like to thank an anonymous reviewer for pressing me to clarify this point.

11 The name PkANN comes from the fact that when the large scale distribution of matter is given in Fourier space (or k-space), it is called the power spectrum and written as *P(k)*.

12 See Bishop (1995) and Batilo (2015) for more on the construction of ANNs.

13 I would like to thank an anonymous reviewer for pointing me to this reference.

14 Consider training an ANN to decide whether to give an applicant a loan. In such a case, one wants clear reasons for the artificial neural network’s decision, reasons that go beyond reference to functional relations in the data set that it was trained on. One ought to be interested in (and perhaps worried about) what structural trends the ANN is basing its decision on. See Creel (2020, [§4]) for a discussion of how transparency might be increased in such a situation. See Sullivan (2019) for further discussion of the use of (deep) neural networks in contexts where there may be a lack of support for the link between the model and the target system.

## References

[CR1] Agarwal S, Abdalla FB (2012). PkANN - I. Non-linear matter power spectrum interpolation through artificial neural networks. Monthly Notices of the Royal Astronomical Society.

[CR32] Agarwal S, Abdalla FB (2014). PkANN – II. A non-linear matter power spectrum interpolator developed using artificial neural networks. Monthly Notices of the Royal Astronomical.

[CR2] Ashby, W. (1956). *An introduction to cybernetics*. University paperbacks: Chapman & Hall.

[CR3] Batilo, A. (2015). *Everything you need to know about artificial neural networks. Medium: technology, invention, app, and more*. https://medium.com/technology-invention-and-more/everything-you-need-to-know-about-artificial-neural-networks-57fac18245a1.

[CR4] Batterman RW (1992). Explanatory instability. Noûs.

[CR5] Batterman RW, Rice CC (2014). Minimal model explanations. Philosophy of Science.

[CR6] Belevitch V (1962). Summary of the history of circuit theory. Proceedings of the IRE.

[CR7] Bishop, C., Hinton, G., Press, O. U., & Bishop, P. (1995). Advanced texts in econometrics. *Neural networks for pattern recognition*. Clarendon Press.

[CR8] Blatt, J., & Weisskopf, V. (1952). *Theoretical nuclear physics*. Wiley.

[CR9] Brandenberger, R. H. (2004). Lectures on the theory of cosmological perturbations. In N. Bretón, J. L. Cervantes-Cota, and M. Salgad (Eds.), *The early universe and observational cosmology*, Volume 646 of *Lecture notes in physics* (pp. 127–167). Springer.

[CR10] Coles, P., & Lucchin, F. (2002). *Cosmology: The origin and evolution of cosmic structure*. Wiley.

[CR11] Creel KA (2020). Transparency in complex computational systems. Philosophy of Science.

[CR12] Gooding DC (1974). Philosophy and science: The black box again. Metaphilosophy.

[CR13] Habib S, Heitmann K, Higdon D (2007). Cosmic calibration: Constraints from the matter power spectrum and the cosmic microwave background. Physical Review D.

[CR14] He S, Li Y, Feng Y (2019). Learning to predict the cosmological structure formation. Proceedings of the National Academy of Sciences.

[CR15] Heitmann K, Higdon D, White M (2009). The coyote universe. II. Cosmological models and precision emulation of the nonlinear matter power spectrum. The Astrophysical Journal.

[CR16] Heitmann K, White M, Wagner C (2010). The coyote universe. I. Precision determination of the nonlinear matter power spectrum. The Astrophysical Journal.

[CR17] Kennedy M, Anderson C, Conti S, O’Hagan A (2006). Case studies in gaussian process modelling of computer codes. Reliability Engineering & System Safety.

[CR18] Kern NS, Liu A, Parsons AR (2017). Emulating simulations of cosmic dawn for 21 cm power spectrum constraints on cosmology, reionization, and x-ray heating. The Astrophysical Journal.

[CR19] Lawrence, E., Heitmann, K., Higdon, D., et al. (2010). Cosmic emulation: The universe as a black box, pp. 50–51.

[CR20] Maxwell JC (1879). Thomson and Tait’s natural philosophy. Nature.

[CR21] Mohammadi, H., Challenor, P., & Goodfellow, M. (2018). Emulating dynamic non-linear simulators using Gaussian processes. ArXiv e-prints.

[CR22] Ntampaka, M., Avestruz, C., Boada, S., et al. (2019). The role of machine learning in the next decade of cosmology. *Bulletin of the AAS*, *51*(3). https://baas.aas.org/pub/2020n3i014.

[CR23] O’Connor, C. (2017). Modeling minimal conditions for inequity. Unpublished manuscript. http://cailinoconnor.com/wp-content/uploads/2015/03/Modeling-Minimal-Conditions-for-Inequitysept2017.pdf.

[CR24] Peebles, P. (1980). *The large-scale structure of the universe. Princeton series in physics*. Princeton University Press.

[CR25] Peter, P., & Uzan, J. (2013). *Primordial cosmology. Oxford graduate texts*. OUP Oxford.

[CR26] Schneider MD, Holm Ó, Knox L (2011). Intelligent design: On the emulation of cosmological simulations. The Astrophysical Journal.

[CR27] Serra-Ricart M, Garrido L, Gaitan V (1994). Statistical methods in astronomy based on artificial neural network techniques. Vistas in Astronomy.

[CR28] Strevens, M. (2016). Special-science autonomy and the division of labor. In M. Couch & J. Pfeifer (Eds.), *The philosophy of Philip Kitcher*. Oxford University Press.

[CR29] Sullivan, E. (2019). Understanding from machine learning models. *The British Journal for the Philosophy of Science*. axz035.

[CR30] Tulin S, Yu H-B (2018). Dark matter self-interactions and small scale structure. Physics Reports.

[CR31] Weisberg M (2007). Three kinds of idealization. Journal of Philosophy.

